# Adolescent-parent communication on sexual and reproductive health issues and its factors among secondary and preparatory school students in Hadiya Zone, Southern Ethiopia: institution based cross sectional study

**DOI:** 10.1186/s12887-018-1388-0

**Published:** 2019-01-07

**Authors:** Samuel Kusheta, Belay Bancha, Yitagesu Habtu, Degefa Helamo, Samuel Yohannes

**Affiliations:** 1 Department of Health Service Extension Program, Hossana College of Health Sciences, Hossana, Ethiopia; 2 School of Public and Environmental Health; department of sexual and reproductive health, Jimma University, Jimma Southwest, Ethiopia

**Keywords:** Adolescent-parent communication, Sexual and reproductive health, Adolescents, Late adolescent, Adolescent and youth friendly services

## Abstract

**Background:**

Adolescent communication with parents is paramount to reduce sexual health problems. Currently, there is a shortage of information on adolescent-parent communication in Ethiopia in general and study area in particular. Thus, this study is intended to determine adolescent-parent communication on sexual and reproductive health issues and its factors among secondary and preparatory school adolescents in Hadiya Zone, Ethiopia.

**Methods:**

We used institution based cross-sectional study design. We stratified schools into urban and semi-urban settings. Then, a total of 8 schools were randomly selected from the strata. The sample size was allocated for each stratum. Finally, participants were randomly selected from separate sampling frames prepared for each stratum. We developed structured questionnaire from related literatures to collect data on adolescent-parent communication and its factors. We cleaned and entered data using EPI info version 3.5.3 and exported to SPSS version 20 for descriptive and logistic regression analysis.

**Results:**

The proportion of adolescents who had communicated with their parents was 144 (35.0%). Multivariate logistic regression analysis indicates that participants’ knowledge about availability of adolescent and youth friendly sexual and reproductive health services at health facilities [AOR: 0.40, 95% CI: (0.26, 0.62),*P-value = 0.001*], utilization of adolescent and youth friendly sexual and reproductive health services [AOR: 0.46, 95% CI: (0.29, 0.72),*P*-value = 0.001] and respondents’ educational status: being grade 9, [AOR: 3.21, (95% CI: ((1.16, 8.89), *P*-value = 0.025] and grade 11; [AOR: 2.96, (95% CI: (1.06, 8.30),*P*- value =0.039] were statistically associated factors affecting adolescents for not communicating with parents on sexual and reproductive health issues.

**Conclusion:**

The findings of our study imply that adolescents were not communicating much with parents about sexual and reproductive health issues even though they were aware of adolescent and youth friendly sexual and reproductive health services. In addition, promotion of service availability may be important to motivate adolescents to communicate with parents. Contextual and age dependent communication barriers should be further identified. Further research is needed in the area to identify barriers particularly from parent side.

## Background

Around 24.5% (1.8 billion) of the world’s population are adolescents and youths aged 10 to 24 years in 2015. Of which, 18% of all adolescents and youths live in Africa. In Ethiopia, more than 35% of the total population is made from adolescents and youths aged 10 to 24 years [1]. This big number is still facing big challenges in sexual and reproductive health and right services. The global community in general; Africa in particular has still a lot of unfinished agenda with regard to major STIs among adolescents. Adolescents, a vulnerable populations have multiple sexual and reproductive health problems including gender inequality, sexual coercion and partner violence, early marriage, polygamy, female genital mutilation, unplanned pregnancies, closely spaced pregnancies, abortion, sexually transmitted infections (STIs) including HIV/AIDS [2–5].

Researches indicate that adolescents in sub-Saharan Africa were not well informed about sexual and reproductive health matters, because their major sources of information are friends. Other informal sources like parents had got low attention while parents could be a key strategy to reach adolescents. Although, parents were themselves often uninformed and preferred that their children learn from teachers or health-care workers, teacher /health care professionals in turn believed that parents should have the primary responsibility for providing information [6, 7].

As long as adolescents are from diverse community, inclusive behavioral interventions are needed that take account of the social context, attempt to modify social norms to support uptake and maintenance of behavior change, and tackle the structural factors that contribute to risky sexual behavior [7]. As evidences suggest, adolescent-parent communication may be one proximate strategy among several strategies that improves healthy sexual and reproductive health behavior [8–10]. The universal access to sexual and reproductive health-care services set by the united nation would not be realized unless we reach adolescents through various interventions including parents in low- and middle-income countries [11, 12].

Adolescents need adults—especially parents, who will connect with them, communicate with them, spend time with them, and show a genuine interest in them. Despite adolescents often have difficulty in communicating about sexuality with their parents; it helps them to establish individual values to healthy sexual behavior [13]. Talking with adolescents about sex-related topics including abstinence, improved contraception, how to prevent HIV and other sexually transmitted infections (STIs) is a positive parenting practice that has been widely researched [13, 14].

Researches have showed that adolescent–parent communication about sexual issues can reduce adolescents’ sexual risk [14, 15]. An HIV/AIDS intervention research established that parent-adolescent communication on sex-related issues improved youth’s condom use skills and self-efficacy [16].Given that parental factors are resolved through helping parents [17], sexual communication with parents, particularly mothers, plays a role in having safer sex behavior [8].

However, adolescent communication about sexual and reproductive health (SRH) issues is affected by social norms and taboos related to gender and sexuality. These factors create a culture of silence, particularly for adolescent girls, in asking, obtaining information, discussing, and expressing their worries about SRH [18]. Similarly, cultural and religious beliefs of parents that adolescents are too young to discuss about sexual issues and unfavorable environment for discussion hinder adolescent-parent communication on sexual health issues [9, 19].

In Ethiopia, evidences show that sex, age, lack of parental interest to discuss, feeling ashamed and cultural unacceptability to talk about sexual matters [19–22] were factors affecting adolescent-parent communication. These may contribute to low utilization of adolescent and youth friendly sexual and reproductive health services (AYFSRHs) in the country.

In Ethiopian context of ethnic and cultural diversity, adolescent-parent communication about sexual and reproductive health issues is an important factor that may reduce engagement in risky sexual behavior. As to our knowledge, there is limited information regarding parent- adolescent communication and its factors among adolescents attending high school**.** Therefore, the objective of the study is to determine adolescent-parent communication on sexual and reproductive health issues and its factors among high school adolescents aged 15 to 19 years old.

## Methods

### Study setting

This study was conducted in secondary and preparatory schools located in Southern Ethiopia; Hadiya Zone. The Zone has a total of 42 secondary schools (35 governmental and 7 private) during the study. Of which, 9 and 33 schools are situated in urban and semi-urban settings, respectively. A total number of students enrolled in all the schools is 60,532 (31,920 males and 28,612 females) in 2016**.**

Despite remarkable progress has been made in increasing access to adolescent-friendly reproductive health services, more efforts are needed to shrink wide range of SRH problems in Ethiopia in general and study area in particular. The strategy that is being currently implemented in the study area lacks focus on adolescent-parent communication particularly on sexuality and sensitive reproductive health issues due to various sociocultural and health service related factors [23].

### Study design and period

We used institution based cross sectional study design from April to August; 2016.

### Study participants

The source population was all adolescents whose age ranges from 15 to 19 years attending their secondary schools. The study population included adolescents (15–19 years old) who had the chance of being randomly selected from the source population. Adolescents whose age is less than 15 years were excluded from the study due to the fact that they might not provide accurate information with regard to sexual and reproductive health issues.

### Sample size calculation

The sample size for the study was estimated by using single population proportion formula at 95% confidence level (CI), Z (1-ά/2) = 1.96), an expected proportion of adolescent-parent communication, 59.1%, from the study conducted in southern Ethiopia [24] and, 5% margin of error. Using the above assumptions, the sample size was calculated as follows.

$$ \mathrm{n}=\frac{\mathrm{Z}{\frac{\upalpha}{2}}^2\mathrm{P}\left(1-\mathrm{P}\right)}{{\mathrm{d}}^2} $$

$$ \mathrm{n}=\frac{{\left(1.96\ \right)}^20.591\left(1-0.591\right)}{(0.05)^2}=372 $$

$$ \kern0.6em =372+372\ \left(15\%\right)=428 $$

We considered none-response rate of 15% in the estimation of the minimum sample size required for the study. Therefore, the final sample size was **428** adolescents in the age group 15–19 years.

### Sampling techniques

First we stratified schools into urban and semi-urban secondary schools. Once we stratified schools, we randomly selected 2 schools from urban and 6 schools from semi-urban areas. Then the calculated sample size was proportionally allocated to randomly selected schools by size of students in each school. Again, we prepared another strata based on students’ grades in the selected schools and proportional sample of students was allocated to each stratum. For each stratum (student’s grade), we prepared sampling frame comprising students whose age ranges from 15 to 19 years. Finally, we randomly selected study participants independently from the prepared sampling frame prepared using lottery method in each stratum until the allocated sample size is reached.

### Data collection and quality procedures

We developed structured questionnaire by reviewing related literatures and previous studies in accordance with the stated objectives of the study. Relevant contents have been extracted from standardized questionnaire developed by John Cleland and included in our questionnaire [25]. The questionnaire was first prepared in English, translated into Amharic and then re-translated back to English to check its consistency. We conducted a pretest on 5% of our sample size among students enrolled in secondary schools (private and government) other than the study setting. We corrected and revised the questionnaire based on the gaps identified during the pretest. We used self-administered data collection technique to gather data. We recruited four supervisors and oriented them how to supervise the data completion procedures. Informed verbal consent from the participants [18 or older age] and assent and informed parental consent (less than 18 years of old) was obtained before the questionnaire have been completed. The completed questionnaire was checked for its consistency and completeness.

### Study variables

Data were collected on independent variables like socio-demographic and economic, socio-cultural, religion, ethnicity, participants’ knowledge about and attitude towards sexual and reproductive health issues, individual/personal factors related to reproductive health service, and other sexual and reproductive health service factors. Adolescent-parent communication about sexual and reproductive health issues/ behavior is a dependent or an outcome variable.

### Operational definitions

#### Parents

Refer to household members of the study participants to encompass WHO definition “all those who provide significant and/or primary care for adolescents, over a significant period of the adolescent’s life, without being paid as an employee,” [17] such as biological parents (father, mother), grandparents, elder sister/brothers and any other caretakers.

#### Parental communication

Refers to a discussion made among the study participants and parents on at least one of the sexual and reproductive health issues (sexuality and sexuality education, prevention of sexually transmitted infections (STI), unintended pregnancy and safe abortion, antenatal care, sexual violence and right and so on) in their life time.

#### Adolescent

Refers to study participants in the age range from 15 to 19 years.

#### Mother/father

Applies for both the students’ biological parents and guardians.

### Data processing and statistical analysis

Data were cleaned and entered to EPI info version 3.5.3 [26] and exported to SPSS version 20 [27] for descriptive and logistic regression analysis. We used descriptive data analysis techniques to describe the distribution of factors for adolescent-parent-communication among adolescents. We employed logistic regression to identify associated factors for adolescent-parent communication about sexual and reproductive health issues. We computed odds ratio with 95% CI to show the strength of the association between the adolescent-parent communication and associated factors. All variables which showed statistically significant results (*P-value < 0.05*) with adolescent-parent communication in bivariate logistic regression were taken to multivariate logistic regression model. Thus, the independent effect of each explanatory variable on an outcome variable was determined while controlled for others.

## Results

### Socio-demographic characteristics of study participants

Out of 428 participants, 411 study participants were considered for analysis which gave the overall response rate of about 96%. The study participants were in the age range of 15 to 19 years with mean age of 17.73 + 1.18 SD years. Among 411 study participants, 210 (51.1%) were males. Majority of the participants, 244 (59.4%), were residents living in the rural parts of the Hadiya Zone and most of them, 287 (69.8%), were living together with their parents. The mean monthly family income of the study participants was 72.70 + 62.93SD USD. Thirty eight (9.2%) of the study participants had their own average monthly income of 11.92 + 10.08 SD USD. The distribution of socio-demographic characteristics of the participants is depicted bellow (Table 1).

**Table 1:** Socio-demographic characteristics of study participants on adolescent- communication about sexual and reproductive health issues in Hadiya Zone, Ethiopia, 2016 (*n* = 411)

Variables	Frequency	Percent
Age group		
Below median age	128	31.1
Above median age	283	68.9
Sex		
Male	210	51.1
Female	201	48.9
Educational level of respondents		
Grade 9	172	41.8
Grade 10	64	15.6
Grade 11	139	33.8
Grade 12	36	8.8
Religion		
Protestant	285	69.3
Orthodox	68	16.5
Catholic	27	6.6
Muslim	22	5.4
Others	9	2.2
Ethnicity		
Hadiya	380	92.5
Kembata	9	2.2
Gurage	13	3.1
Others^a^	9	2.2
Parents permanent residence		
Urban	167	40.6
Rural	244	59.4
Students’ current residence		
Urban with family	140	34.1
Urban house rent	126	30.7
Coming from rural daily	145	35.1
Marital status		
Single	392	95.4
Married	14	3.4
Divorced	3	0.7
Widowed	2	0.5
Students’ mother education^b^		
Illiterate	187	45.5
Read and write	39	9.5
Primary education(1–6 grade)	68	16.5
Junior secondary(7–8 grade)	49	11.9
Secondary education(9–12 grade)	44	10.7
Higher education (12+ grade)	24	5.8
Students’ father education^b^		
Illiterate	121	29.4
Read and write	62	15.1
Primary education(1–6 grade)	45	10.9
Junior secondary(grade (7–8 grade)	57	13.9
Secondary education(9–12 grade)	51	12.4
Higher education (12+ grade)	75	18.2
Students’ parent monthly income		
< $26	103	25.1
$27–52	131	31.9
> $52	177	43.1
Students’ monthly income status		
No	373	90.8
Yes	38	9.2

Others^a^Silte, Wolayita, Gamo, Amhara, ^b^refers biological mother /father and/ or caretaker

### Adolescent –parent communication about sexual and reproductive health issues and risk behaviors

The proportion of adolescents who had communicated with their parents regarding sexual and reproductive health issues was 144 (35.0%). Of which, females represent 75(52.1%) followed by males 69 (47.9%). Participants’ brothers were the most preferred family members 46 (11.2%) by adolescents to communicate with about sexual and reproductive health issues followed by fathers which accounted for 42 (10.2%). Of the 144 adolescents, 123 (85.4%), 115(79.9%) and 70 (48.6%) had accounted for HIV counseling and testing, contraception and/or condom, and sexually transmitted infections respectively. Seventy nine (19.2%) of participants had experienced sexual intercourse at least once in their life time. The mean age at which they experienced their first sexual intercourse was 15.89 + 1.57 SD years. Among respondents who had sexual intercourse, self-sexual-drive was the leading, 54 (68.4%) motivator for the adolescents to engage in sexual activity followed by peer pressure 36 (45.6%). Thirty three (8.0%) of the study participants used at least one substance. Among these, 17 (51.5%) of substance users had been using khat followed by alcohol, 12(36.4%) (Table 2).

**Table 2:** Adolescent-parent communication among respondents about sexual and reproductive health issues in Hadiya Zone, Ethiopia, 2016

Variables	Frequency	Percent
Ever had parental communication about sexual and reproductive health issues (*n* = 411)		
No	267	65.0
Yes	144	35.0
Most preferred members to communicate with (*n* = 144)		
Father	42	10.2
Mother	26	6.3
Brother	46	11.2
Sister	26	6.3
Other(uncle, aunt)	4	1.0
Communication themes with parents (*n* = 144)^a^		
Contraception and / or condom	115	79.9
HIV counseling and testing	123	85.4
Sexually transmitted infections	70	48.6
Screening and treatment seeking	52	36.1
Pregnancy test	64	44.4
Unwanted pregnancy and/or safe abortion	26	18.1
Sexual engagement	20	13.9
Others^b^	19	13.2
I don’t remember	2	1.4
Ever had sexual intercourse (*n* = 411)		
No	332	80.8
Yes	79	19.2
Motivation for sexual intercourse (*n* = 79)^a^		
Self-drive	54	68.4
Peer pressure	36	45.6
Alcohol	10	12.7
Substances (khat)	6	7.6
Do you have children (*n* = 79)		
No	69	87.3
Yes	10	12.7
History of reproductive health problems (*n* = 79)		
No	57	72.2
Yes	22	27.8
Reproductive health problems (*n* = 22)^a^		
Unwanted pregnancy	12	54.5
Abortion and /or complication	9	40.9
Sexually transmitted infection	6	27.3
Sexual coercion	7	31.8
Ever used substances (*n* = 411)		
No	378	92.0
Yes	33	8.0
Type of substances used (*n* = 33)		
Khat	17	51.5
Alcohol	12	36.4
Tobacco	9	27.3
‘Shisha’	2	6.1

^a^Indicates multiple response**,** ^b^Includes like sexual engagement, menstruation, gender violence, sexual rights

### Individual factors on sexual and reproductive health services

Majority, 330 (80.3%), of the participants had information about adolescent and youth friendly sexual and reproductive health services (AYFSRHs). However, less than half, 179 (43.6%) of the participants had information about availability of AYFSRHs at nearest health facilities/youth centers. Among participants who had information, 229 (69.4%), 209 (63.3%) and 98 (29.7%) had been informed about HIV/AIDS counseling and testing, contraception and/or condom services, and prevention of sexually transmitted infections respectively. Of the participants who had been informed about AYFSRH, 291 (88.2%) believed that utilization of the service promotes healthy sexual and reproductive behavior. Seventy (17.0%) of study participants reported that they did not get AYFSRHs from the nearest health facilities/youth centers despite of their intention to use. The two major reasons cited for not using the services were inconvenient service time, 19(27.1%), followed by long waiting time, 16(22.9%) (Table 3).

**Table 3:** Knowledge about and attitude towards sexual and reproductive health issues among study participants in Hadiya Zone, Ethiopia, 2016

Variables	Frequency	Percent
Ever had heard about adolescent and youth friendly sexual and reproductive health services (AYFSRHs) (*n* = 411)		
No	81	19.7
Yes	330	80.3
Youth friendly sexual and reproductive health services mentioned by participants (*n* = 330)^a^		
HIV/AIDS counseling and testing	229	69.4
Contraception and / or condom services	209	63.3
Prevention of sexually transmitted infections	98	29.7
Screening and treatment of STI	70	21.2
Antenatal care for young pregnant persons	67	20.3
Pregnancy test	58	17.6
Other sexual and reproductive health issues	10	3.0
I don’t remember	4	1.2
Perceived benefit adolescent- parent communication about sexual and reproductive health (*n* = 330)		
No	39	11.8.
Yes	291	88.2
Ever had information about availability of AYFSRHs at any health facilities		
No	232	56.4
Yes	179	43.6
Major information source (*n* = 179)^a^		
Peers	98	54.7
Parent and /or guardian	53	29.6
Teacher	10	5.6
Mass media	19	10.6
Ever used AYFSRHs		
No	260	63.3
Yes	151	36.7
AYFSRHs utilized by participants (*n* = 151)^a^		
HIV Counseling and testing	93	61.6
Contraception and/ or condom	64	42.4
Antenatal care services	10	6.6
Treatment of STI	23	15.2
Safe and /or post abortion care	6	4.0
Pregnancy testing	7	4.6
Have you ever missed SRHS when visiting any AYFSRHs		
No	341	82.7
Yes	70	17.0
Cited reasons for missing AYFSRHs (*n* = 70)^a^		
Inconvenient time of the service	19	27.1
Long waiting time	16	22.9
Lack money to purchase the service	14	20.0
Feeling of shame	12	17.1
Lack of privacy at the facility	15	21.4
The service provider refused to give the service	13	18.6
The service unit is closed at a health facility	15	21.4

^a^Indicates multiple response

### Factors associated with adolescent-parent communication about sexual and reproductive health issues

As observed from multivariate logistic regression analysis, participants’ knowledge about the service availability, utilization of AYFSRHs and respondents’ education were significantly associated with adolescent-parent communication. Those who had no information about availability of AYFSRHs at health facilities were 60% less likely to communicate with their parents about sexual and reproductive health issues [AOR: 0.40, 95% CI: (0.26, 0.62), *P*-value = 0.001] than those who had. Likewise, those who had not utilized AYFSRHs were about 54% less likely to communicate with their parents when compared to those who had utilized the service [AOR: 0.46, 95% CI: (0.29, 0.72), *P*-value = 0.001]. Lower grade (grade 9) participants were about 3 times more likely to communicate about sexual and reproductive issues as compared to higher grades participants (grade twelve and above) [AOR: 3.21, 95%CI: (1.16, 8.89),*P*- value = 0.025]. Moreover, participants from grade 11 were about 3 times more likely to communicate than those from grade twelve and above [AOR: 2.96, 95%CI: (1.06, 8.30), *P*- value =0.039] (Table 4).

**Table 4:** Factors associated with parent-adolescent communication about sexual and reproductive health issues among the respondents in Hadiya Zone, Ethiopia,2016

Variables	Frequency	Crude OR (COR)		Adjusted OR(AOR)	
		95% CI	*P* value	95% CI	*P* value
Age group					
Below median age	128	1.23 (0.84, 1.99)	0.250		
Above median age	283	1			
Sex					
Male	210	1.22(0.81, 1.82)	0.344		
Female	201	1			
Religion					
Protestant	285	1			
Orthodox	68	0.52 (0.19,1.47)	0.221		
Catholic	27	0.95 (0.54, 1.65)	0.844		
Muslim	22	2.20(0.83, 5.84)	0.113		
Others	9	2.29(0.56,9.35)	0.248		
Respondent’s grade^a^					
Grade 9	172	4.46 (1.66,12.04)	0.003	3.21 (1.16, 8.89)	0.025
Grade 10	64	1.43 (0.46, 4.45)	0.536	1.07 (0.33, 3.48)	0.913
Grade 11	139	4.06(1.49, 11.08)	0.006	2.96 (1.06,8.30)	0.039
Grade 12	36	1		1	
Family residence					
Urban	167	1			
Rural	244	0.89 (0.59,1.35)	0.60		
Students’ current residence					
Urban with family	140	1			
Urban house rent	126	1.18 (0.72, 1.95)	0.505		
Coming from rural daily	145	0.78(0.48, 1.29)	0.336		
Students’ mother education					
Illiterate	187	1			
Read and write	39	0.77 (0.36,1.65)	0.507		
Primary school (1–6)	68	0.88 (0.48,1.59)	0.673		
Junior secondary school (7–8)	49	1.36 (0.71,2.59)	0.353		
Secondary school (9–12)	44	1.64 (0.84,3.19)	0.146		
Higher education (12+)	24	1.18 (0.94,2.85)	0.711		
Students’ father education					
Illiterate	121	1			
Read and write	62	1.75(0.93,3.30)	0.083		
Primary school (1–6)	45	1.51(0.74, 3.08)	0.253		
Junior secondary school (7–8)	57	1.23 (0.63, 239)	0.548		
Secondary school (9–12)	51	1.35 (0.68, 2.69)	0.394		
Higher education (12+)	75	1.00(0.54, 1.88)	0.990		
Students’ parent monthly income					
< $26	103	0.66(0.39, 1.12)	0.121		
$27–52	131	1.00(0.63,1.60)	0.983		
> $52	177	1			
History of sexual intercourse					
No	332	1			
Yes	79	1.33(0.90,1.95)	0.148		
Ever had heard about AYFSRHs					
No	81	1.15 (0.68,1.85)	0.674		
Yes	330	1			
Knowledge about availability of AYFSRHs at health facilities^a^					
No	232	0.33(0.22, 0.50)	0.001	0.40(0.26,0.62)	0.001
Yes	179	1		1	
Ever used AYFSRHs^a^					
No	260	0.38 (0.25, 0.58)	0.001	0.46(0.29, 0.72)	0.001
Yes	151	1		1	
AYFSRH visiting status in the last six month					
No	341	0.58 (0.35, 0.98)	0.041	0.85(0.49,1.49)	0.575
Yes	70	1		1	

^a^significantly associated factors

## Discussion

The practice of risky sexual behaviors of adolescents results in sexually transmitted infections, unintended pregnancies, poor sexual engagement and delayed or absence of early management of adverse outcomes of sexuality and reproductive health problems. Evidences suggest that adolescent-parent communication about sexuality and reproductive health issues helps adolescents avoid the experience of such a risky sexual and reproductive health behaviors. Therefore, this study addresses adolescent-parent communication and factors associated with sexual and reproductive health issues among adolescents attending their secondary and preparatory schools. Consequently, it might give insights to the policy programmers for the development of appropriate interventions.

Our study presents that only 35.0% of adolescents had communicated with their parents. This finding is less than the findings of the studies conducted in Debremarkos, Northwest Ethiopia, 36.9% [28], Sidama Zone, Southern Ethiopia, 59.1% [24], Dire Dawa, East Ethiopia, 37% [21] and Mekele, North Ethiopia, 43.5% [29]. This could be due to the fact that majority of the study participants (59.4%) in our study reside in rural settings which might have reduced their exposure to sexual and reproductive health information and this might have subsequently reduced the opportunity to communicate with their parents.

In this study, adolescent-parent communication is nearly two folds lower than the finding from hospital based study on condom use, 76%, in America [30]. This could be explained by limited exposure of adolescents and parents to reproductive health information to utilize the services in less developed nations when compared to those in well developed nations. In addition, as evidenced by a literature [31], low level of parental connectedness as a result of low awareness about sexual and reproductive health issues and lack of close supervision contribute for low level of parental communication.

However, our finding is slightly greater than the study findings from northwest Ethiopia, Awabel Woreda, 25.3% [20], west Ethiopia, East Wollega Zone, 32.5% [22] and east Ethiopia, Harar 28.8% [32] and India, 29% [33]. The observed difference could be due to the fact that our study is more recent than the aforementioned studies in which both parents and adolescents might have had better access to promotional activities about sexual and reproductive health information. With regard to communication preferences, participants’ brothers were the most preferred to other members of the family about sexual and reproductive health issues. This finding contradicts with the finding from northwest Ethiopia, Awabel Woreda [20] where most of the participants, 31.4% preferred mothers to other members of their family to communicate with about reproductive health matters.

Many school based interventions have been realized on the ground such as family planning, prevention and control of STI, HIV/AIDS, and gender violence and so on in Ethiopia. On the contrary to the available interventions, our finding implies that adolescent-parent communication had low achievement. Though several factors might contribute for the low achievement, parental factors played a role for low adolescent-parent communication as stated in the study done in Ethiopia [29] and Uganda [34]. Thus, the Ethiopian adolescent and youth friendly service programs should have to go a long distance to achieve better coverage in adolescent-parent communication.

As observed in our descriptive analysis, 19.7% of adolescents had not ever heard about AYFSRHs. This figure is lower than the study done in Debremarkos, northern Ethiopia [28] which accounted for, 37.6%. More than half, 56.4% of study participants reported that they had never been informed about the availability of adolescent and youth friendly sexual and reproductive health services in the health facilities. Whatever the case, the finding indicated that there had been poor promotional activities about adolescent and youth friendly services in schools with the intention to reach adolescents. As stated by the World Health Organization [7, 9] school based sexual and reproductive health promotion is vital in reaching adolescents.

The practice of risky sexual behavior had been analyzed for the study participants. Accordingly, 19.2% of the study participants were sexually active at the mean age of 15.89 + 1.57 SD years during their first sexual intercourse. Our finding indicated that participants had earlier sexual experience when compared to the study finding in Gamo Gofa [35], but delayed than the finding from the study in Debremarkos, North Ethiopia [28]. Eight percent of the study participants abused at least one substance. This finding is less than the study finding in Bale, South West Ethiopia, 34.8% [36], Woreta Town, Northwest Ethiopia, 47.9% [37] and Harar, East Ethiopia [32]. This might be due to cultural differences and variations in associated interventions with respect to sexual and reproductive health problems among the study settings.

Those adolescents who had no information about availability of AYFSRHs at health facilities were less likely to communicate with their parents about sexual and reproductive health issues. This is supported by the finding from the study in Debremarkos, North Ethiopia [26]. This could be due to the fact that absence of information about the availability of the service might have influenced the ability of the adolescents to communicate with their parents. Regarding the service utilization, those adolescents who had not utilized AYFSRHs were less likely to communicate with their parents when compared to those who had utilized the service. This could be reasoned out that adolescents who had experience in utilizing AYFRHs might have been informed more about the importance of parental communication.

Lower grade (grade 9) participants were more likely to communicate about sexual and reproductive issues as compared to higher grades (twelve and above). Our finding was in line with that of Debremarkos, North Ethiopia [26]. Whereas, it disagrees with the findings in Awabel, North Western Ethiopia [20], Mekele, Northern Ethiopia [28] and Harar, Eastern Ethiopia [32]. This might be due to differences in culture and implementation of school-based sexual and reproductive health interventions. In our study other socio demographic factors were not statistically significant with parent-adolescent communication about sexual and reproductive health issues (Table 4). However, sex and age of the adolescents were factors influencing adolescent parent communication in other studies [19–22].

## Conclusion

The findings of our study imply that adolescents were not communicating much with parents about sexual and reproductive health issues even though they were aware of adolescent and youth friendly sexual and reproductive health services. In addition, promotion of service availability may be important to motivate adolescents to communicate with parents. Contextual and age dependent communication barriers should be further identified. Further research is needed in the area to identify barriers particularly from parent side.

### Limitation of the study

Our study did not address the parent side factors for adolescent-parent communication on sexual and reproductive health issues.

## Abbreviations

AIDS: Acquired Immune Deficiency Syndrome
AYFRHs: Adolescent and Youth Friendly Reproductive Health Services
HIV: Human Immune Deficiency Virus
SNNPR: South Nations, Nationalities and People Regional State
SRH: Sexual and Reproductive Health
